# Variable antibiotic susceptibility patterns among *Streptomyces *species causing actinomycetoma in man and animals

**DOI:** 10.1186/1476-0711-10-24

**Published:** 2011-06-06

**Authors:** Mohamed E Hamid

**Affiliations:** 1 Department of Microbiology, College of Medicine, King Khalid University, P. O. Box 641, Abha, Kingdom of Saudi Arabia

**Keywords:** Antibiotic susceptibility, *Streptomyces*, Actinomycetoma, Sudan

## Abstract

**Background:**

Drug therapy is recommended in conjunction with surgery in treatment of actinomycetoma. The specific prescription depends on the type of bacteria (actinomycetoma) or fungi (eumycetoma) causing the disease and their *in vitro *antimicrobial susceptibility.

**Objectives:**

To investigate the antimicrobial susceptibility among isolates of *Streptomyces *spp. isolated from cases of actinomycetoma in man and animals in Sudan.

**Methods:**

*Streptomyces *strains (n = 18) isolated from cases of actinomycetoma were tested *in vitro *against 15 commonly prescribed antibacterial agents using MIC agar dilution method as per standard guidelines.

**Results:**

*Streptomyces *strains isolated from actinomycetoma fall into various phenotypic groups. All of the strains were inhibited by novobiocin (8 μg/mL), gentamycin (8, 32 μg/mL) and doxycycline (32 μg/mL). Fusidic acid (64 μg/mL) inhibited 94.4% of the strains; bacitracin, streptomycin, cephaloridine, clindamycin, ampicillin, rifampicin and tetracycline (64 μg/mL) inhibited between 61.1 and 77.8% of the strains. All strains were found resistant to amphotericin B (64 μg/mL), penicillin (20 μg/mL) and sulphamethoxazole (64 μg/mL).

**Conclusions:**

Saprophytic *Streptomyces *spp. cause actinomycetoma in man and animal belong to separate phenotypes and have a wide range of susceptibility patterns to antimicrobial agents, which pose a lot of difficulties in selecting effective *in vivo *treatment for actinomycetoma.

## Background

Actinomycetoma is a slowly progressive, destructive infection of the cutaneous and subcutaneous tissues, fascia, and bones, caused by fungi (eumycetoma) or by aerobic actinomycete bacteria (actinomycetoma or actinomycotic mycetoma) and mainly prevalent in tropical countries [1]. The major agents of aerobic actinomycetes are: *Nocardia brasiliensis*, *Actinomadura madurae *and *Streptomyces somaliensis *[2,3]. *Strep. sudanensis *has recently being described as one of the etiological agents of actinomycetoma [4]. Actinomycetoma is a major health problem in parts of Sudan [5-8].

Diagnosis of mycetoma relies on direct examination of grains and isolation of the etiologic agents. The discharging grains represent aggregates of bacterial filaments or fungal hyphae. The salient features of the grains may assist in the clinical diagnosis: eumycetomas due to *Madurella *spp. typically produce black grains; actinomycetomas never produce dark grains, and usually are yellow to orange; and those caused by *Actinomadurae pelletieri *are red to pink [3,7,9]. Analysis of mycetoma sampled for further histological processing provides some clues to the potential microorganism, but culture is the gold standard for diagnosis [10].

It seems likely that patients who do not respond to standard therapeutic treatment may be infected with unknown actinomycetes and thereby requires specific antibiotic treatment regimes [11]. This is a serious problem as actinomycetoma becomes dangerous to health, or even life, when treatment is inadequate or delayed. A thorough microbiological diagnosis while desirable is not always possible due to difficulties in isolating and characterizing the causal agents.

The aim of this study was to investigate the susceptibility of various phenotypes of streptomycetes isolated from cases of actinomycetoma in human (madura foot) and donkeys (fistulus wihters) in Sudan.

## Materials and methods

### Strains

The present study perform antimicrobial sensitivity testing on *Streptomyces *strains (n = 18) isolated from cases of actinomycetoma in human (madura foot) and actinomycetoma in donkeys (fistulus wihters) between 1998 and 2003 in Khartoum State, Sudan. The 18 *Streptomyces *strains are labeled as SD551, SD552, SD559, SD572, SD573, SD575, SD576 (donkey isolates) and *S. somaliensis *DSM 40738^T^, *S. sudanensis *DSM 41923^T^, SD509, DSM41607, DSM41608, DSM41609, Streptomyces spp.: SD511, SD524, SD528, SD534 and DSM40760 (human isolates).

*S. somaliensis *DSM 40738^T ^and *S. sudanensis *DSM 41923^T ^served as controls. Details of strains and isolation methods have been described previously [4,12]. The strains were sub cultured from frozen 20% glycerol stocks on Tryptic Soy agar plates (TSA; Difco). The inoculated plates were incubated aerobically at 37°C for up to seven days.

### Identification scheme

**Figure 1:**
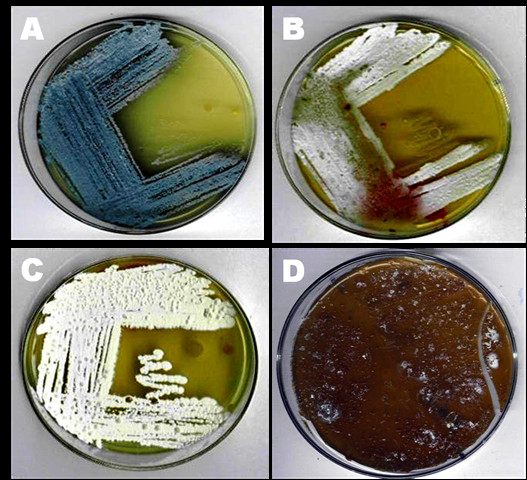
***Streptomyces *spp. exhibiting different phenotypic features: A, *Streptomyces *sp. SD575; B, *Streptomyces *sp. SD579; C, *Streptomyces *sp. SD573 and D, *Streptomyces *sp. SD534**.

Isolates were tentatively identified as member of the genus *Streptomyces *on the basis of selected phenotypic criteria [13]. The cultural and microscopic features of the genus *Streptomyces *are: aerobic growth, gram-positive, non-acid-alcohol-fast, non-motile actinomycete which forms extensively branched, light yellow substrate mycelia on a variety of media with or without aerial hyphae, with or without diffusible pigments on the media. In the present study phenotypic clusters of isolates were identified mainly on colony color and presence of diffusible pigments (Figure 1).

### Antimicrobial Susceptibility Tests

*Streptomyces *isolates (n = 18) including clinical isolates and type strains of *S. somaliensis *DSM 40738^T^, *S. sudanensis *DSM 41923^T ^(Table 1) were tested for their ability to grow in TSA supplemented with antibiotics or antimicrobial agents using the minimum inhibitory concentration (MIC) agar dilution method. These were performed according to standard methods [14]. A homogeneous bacterial suspension giving an inoculum of 10^6^-10^8 ^CFU/mL was prepared by dissolving 1-3 colonies in sterile 2 mL normal saline. The suspension was used to inoculate each TSA plates containing the antimicrobial agents and control plates (without drugs) using a Steer's replicator.

**Table 1:** The results of antimicrobial susceptibility of 18 strains of *Streptomyces *to 15 antimicrobial agents (25 total folds)

Strain* Resistance to antimicrobial agents (μg ml-1):	1	2	3	4	5	6	6	8	9	10	11	12	**13**	14	15	16	17	18
Amphotericin B (16)	R	R	R	R	R	R	R	R	R	R	R	R	R	R	R	R	R	R

Amphotericin B (32)	R	R	R	R	R	R	R	R	R	R	R	R	R	R	R	R	R	R

Amphotericin B (64)	R	R	R	R	R	R	R	R	R	R	R	R	R	R	R	R	R	R

Ampicillin hydrochloride (8)	R	R	R	R	R	R	R	R	R	R	R	R	R	S	R	S	R	R

Ampicillin hydrochloride (32)	R	R	R	R	S	S	R	S	S	S	S	S	S	S	R	S	S	R

Amoxicillin (32)	R	R	R	R	R	S	R	R	R	R	R	R	R	S	R	S	R	R

Amoxicillin (64)	R	R	R	R	R	R	R	R	R	R	R	R	R	R	R	S	R	R

Bacitracin (16)	S	S	R	S	S	S	R	S	S	S	S	S	S	R	S	S	S	R

Cephaloridine hydrochloride (32)	R	R	R	S	S	S	R	S	S	S	S	S	S	S	R	S	S	R

Clindamycin hydrochloride (8)	R	R	R	R	S	S	R	S	S	S	S	S	S	S	R	S	S	R

Doxycycline hydrochloride (16)	S	R	R	S	S	S	S	S	S	S	S	S	S	S	S	S	S	R

Doxycycline hydrochloride (32)	s	s	S	s	s	s	S	S	S	S	S	S	S	S	S	S	S	S

Fusidic acid (32)	S	R	R	S	S	S	R	S	S	S	S	S	S	S	S	S	S	S

Fusidic acid (64)	s	s	R	s	s	s	S	S	S	S	S	S	S	S	S	S	S	S

Gentamycin sulphate (8)	S	S	S	S	S	S	R	S	S	S	S	S	S	S	S	S	S	S

Gentamycin sulphate (32)	s	s	S	s	s	s	S	S	S	S	S	S	S	S	S	S	S	S

Novobiocin (4)	S	R	R	S	S	S	S	S	S	S	S	S	S	S	S	S	S	S

Novobiocin (8)	s	s	S	s	s	s	S	S	S	S	S	S	S	S	S	S	S	S

Rifampicin (32)	R	R	R	S	S	S	R	S	S	S	S	S	S	R	S	S	R	R

Penicillin G (10)	R	R	R	R	R	R	R	R	R	R	R	R	R	R	R	R	R	R

Penicillin G (20)	R	R	R	R	R	R	R	R	R	R	R	R	R	R	R	R	R	R

Streptomycin sulphate (8)	S	R	R	R	S	S	S	S	S	S	S	S	S	R	R	S	S	R

Sulphamethoxazole (32)	R	R	R	R	R	R	R	R	R	R	R	R	R	R	R	R	R	R

sulphamethoxazole (64)	R	R	R	R	R	R	R	R	R	R	R	R	R	R	R	S	R	R

Tetracycline hydrochloride (64)	R	R	R	R	S	S	R	S	S	S	S	S	R	S	S	S	S	R

Abbreviations: S, sensitive, R, resistant.

* Strains: 1, *Streptomyces *sp. SD551; 2, *Streptomyces *sp. SD552; 3, *Streptomyces *sp. SD559; 4, *Streptomyces *sp. SD572; 5, *Streptomyces *sp. SD573; 6, *Streptomyces *sp. SD575; 7, *Streptomyces *sp. SD576; 8, *S. sudanensis *DSM41923^T^; 9, *S. sudanensis *SD509; 10, *S. sudanensis *DSM41607; 11, *S. sudanensis *DSM41608; 12, *S. sudanensis *DSM41609; 13, *S. somaliensis *DSM40738^T^; 14, *Streptomyces *sp. SD 511; 15, *Streptomyces *sp. SD534; 16, *Streptomyces *sp. SD528; 17, *Streptomyces *sp. SD524; 18, *Streptomyces *sp. DSM40760.

A total of 15 antimicrobial agents (Table 1) in twofold to threefold dilution series from 4 to 64 μg/mL making up a total of 25 concentrations were used. The range is within the values recommended by the NCCLS [14]. The test and control agar plates were incubated aerobically at 30°C, for 48-72 h. After incubation, the organisms were classified as sensitive (S) or resistant (R) according to guidelines [14].

## Results

The identification of isolates to cluster and phenotypic groups was done according to microscopic and growth characteristics. The isolates exhibited different phenotypic features (Figure 1). Figure 1 showed examples of colony morphology variations which ranged from grey to blue to grey brown or grey white colonies. The full identification of the isolates is under studies to establish their taxonomic positions.

**Figure 2:**
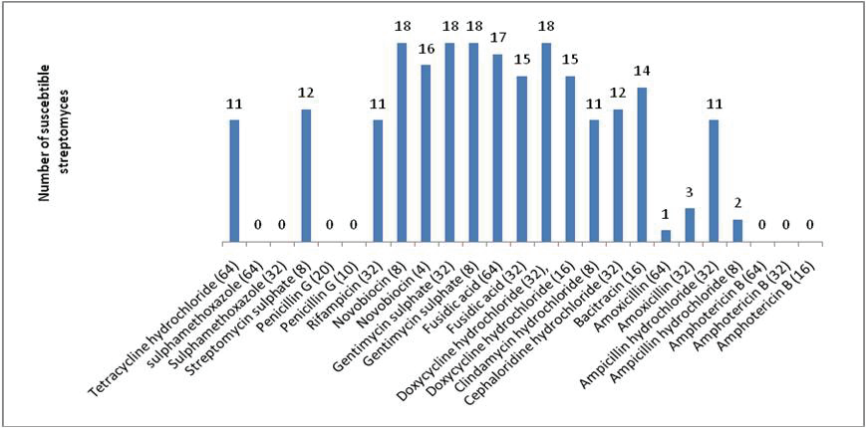
**Susceptibility of different *Streptomyces *spp. causing actinomycetoma to various antimicrobial agents**.

The results of antimicrobial susceptibility testing are shown in Table 1 and Figure 2. Three agents: novobiocin (8 μg/mL), gentamycin sulphate (8, 32 μg/mL) and doxycycline hydrochloride (32 μg/mL) inhibited all strains. Fusidic acid (64 μg/mL) inhibited 94.4% of the strains; bacitracin (16 μg/mL) inhibited 77.8% of the strains; streptomycin sulphate (8 μg/mL) and cephaloridine HCl (32 μg/mL) inhibited 66.7% of the strains; clindamycin HCl (8 μg/mL); ampicillin HCl (32 μg/mL); rifampicin (32 μg/mL) and tetracycline HCl (64 μg/mL) inhibited 61.1% of the strains.

Other antibacterial agents revealed results ranging from 0 to 85%. All strains were found resistant to amphotericin B (64 μg/mL), penicillin (20 μg/mL) and sulphamethoxazole (64 μg/mL) (Table 1; Figure 2).

## Discussion

Regular surveillance of hospital-associated actinomycetoma infections and monitoring of antibiotic sensitivity pattern is required to prompt the treatment alone or in conjunction with surgery to reduce chance of amputation and prolong patient hospitalization due to actinomycetoma. Uses of antibiotic sensitivity testing helps to guide physicians in choosing antibiotics, but our findings indicated that large, seemingly unlimited numbers of saprophytic *Streptomyces*, potentially new species, exhibited various antimicrobial profiles. In the present study 13 phenotypic groups become apparent out of 18 clinical isolates and showed 15 different antibacterial susceptibility profiles. The accumulated sensitivity results on different pathogens guide physicians in choosing empirical treatment in serious patients before the individual's laboratory results are analyzed in the microbiology laboratory. In the present study four antibiotics (novobiocin, gentamycin, doxycycline and fusidic acid) inhibited more than 90% of the strains, which could be alone or in combination one of the choices in the treatment of actinomycetoma. Even though, a combination of sulphamethoxazole and trimethoprim is commonly used as treatment worldwide [15]. Currently the common drugs regimes practiced in Sudan for the treatment of actinomycetoma are amikacin in combination with co-trimoxazole and in resistant cases other drugs such as streptomycin combined with co-trimoxazole or streptomycin combined with rifampicin are practiced [16].

Nasher et al. [17] reported that rifampicin was the most effective antibiotic against *S. somaliensis *strains isolated from Sudanese patients, followed by erythromycin, tobramycin, fusidic acid and streptomycin sulphate. In contrast they found that *S. somaliensis *strains were all resistant to trimethoprim. The present study emphasized that isolates from Sudanese patients are not all *S. somaliensis*, instead *S. sudanensis*, and other unidentified isolates are causal agents with different antimicrobial profiles. The two studies applied different methods and the species analyzed in Nasher et al. [17] study might have included strains other than *S. somaliensis*. Nevertheless, the findings in the present study found that the majority of the strains were sensitive to fusidic acid.

Kelly et al. [18] documented an excellent correlation when four methods for testing the antibacterial, including the agar dilution method which has been adopted in their study [18]. The method was found suitable for testing strains of *Streptomyces *and other clinically significant aerobic actinomycetes. Application of genotypic and phenotypic analysis enables description of many novel species. These are in addition to *S. somaliensis*, the classic known cause of actinomycetoma in human. The slow and often filamentous growth of actinomycetes does not allow sufficient standardization of both the agar dilution and broth dilution method [13]. Knowledge on the antibiotic susceptibility of pathogenic actinomycetes is considered a challenge. This is why the chemotherapy of diseases caused by actinomycetes may still present problems. Complete and reliable data on the *in vitro *sensitivity of these pathogens can be obtained, however, when standardized and specially adapted methods of susceptibility testing are employed [19]. Special adaptations should cover the general technique of *in vitro *testing, as well as the choice of test media, the preparation of inocula, incubation methods and method of reading results. Provided that all factors are taken into account which might cause difficulties in test reproducibility and therapeutic relevance of the results, *in vitro *tests appear to contribute to the revision and updating of treatment regimes for many infections including human actinomycosis [19].

## Conclusions

*Streptomyces *spp. from cases actinomycetoma in man and animals are phenotypically diverse and have wide range of susceptibility patterns to antimicrobial agents. This causes a lot of difficulties in selecting effective *in vivo *treatment for actinomycetoma in man and animals. Studies are underway in Sudan, UK, Poland and Mexico to complete the genotypic characterization of all recognized phenotypes isolated from actinomycetoma cases in Sudan so far.

## Competing interests

The author declares that he has no competing interests.

## Authors' contributions

The author has designed the study, perform the phenotypic characterization, susceptibility testing and have drafted the manuscript. Assistances during field collection of specimens and technical laboratory work are acknowledged in the acknowledgement section.
